# Hydrogen-Positive Small Intestinal Bacterial Overgrowth (SIBO) in Japanese Patients With Chronic Intestinal Pseudo-Obstruction (CIPO): A Cross-Sectional Study

**DOI:** 10.7759/cureus.94054

**Published:** 2025-10-07

**Authors:** Takanori Igarashi, Kentaro Tominaga, Kunihiko Yokoyama, Takuya Wakabayashi, Yuichi Kojima, Yuzo Kawata, Naruhiro Kimura, Kazuya Takahashi, Akira Sakamaki, Shuji Terai

**Affiliations:** 1 Division of Gastroenterology and Hepatology, Graduate School of Medical and Dental Sciences, Niigata University, Niigata, JPN

**Keywords:** breath tests, chronic intestinal pseudo‑obstruction, hydrogen, intestinal methanogen overgrowth, methanogen, small intestinal bacterial overgrowth

## Abstract

Background: Evidence for small intestinal bacterial overgrowth (SIBO) in chronic intestinal pseudo‑obstruction (CIPO) remains limited in Asian populations and by gas phenotype. This study aimed to determine the prevalence and gas phenotype distribution (hydrogen-positive SIBO vs. intestinal methanogen overgrowth) in Japanese patients with CIPO using glucose breath testing and to describe associated clinical features.

Methods: This single‑center cross‑sectional study was conducted at Niigata University, Niigata, Japan (April 2019-March 2022), and included 10 CIPO outpatients and 10 healthy controls. Participants fasted for 12 hours and avoided high-fiber foods for 24 hours before testing. Glucose breath testing (50 g, institutional SOP) measured hydrogen (H₂) and methane (CH₄); positivity followed North American Consensus criteria (ΔH₂ ≥20 ppm by 90 min; CH₄ ≥10 ppm). The primary outcome was hydrogen‑positive SIBO (H₂‑SIBO); intestinal methanogen overgrowth (IMO) was analyzed separately. Exact tests accounted for the small sample size.

Results: H₂‑SIBO was more frequent in CIPO than in controls (5/10 vs. 0/10; Fisher two‑sided p = 0.0325; continuity‑corrected OR = 21). Overall SIBO (H₂ or CH₄) was 5/10 vs. 1/10 (p = 0.141, two‑sided). No CIPO patient met IMO criteria; one control was methane‑positive. Among CIPO patients, H₂‑SIBO was associated with lower BMI and a higher frequency of diarrhea.

Conclusions: In this small Japanese cohort, CIPO was associated with H₂‑SIBO, whereas IMO was uncommon. These findings should be interpreted with caution due to limited statistical power and potential under-detection of distal overgrowth with glucose substrate. These findings may inform on diagnostic strategies and nutritional management in severe motility disorders.

## Introduction

Chronic intestinal pseudo‑obstruction (CIPO) is a rare, debilitating motility disorder first described by Dudley et al. in 1958 [1]. It is characterized by recurrent symptoms of intestinal obstruction without any mechanical cause. Although its pathophysiology remains incompletely understood, CIPO is increasingly recognized as a cause of chronic malabsorption and intestinal failure [2]. Severe impairment of gut motility predisposes to small intestinal bacterial overgrowth (SIBO), which can further exacerbate malnutrition and dysmotility [3,4].

Emerging evidence suggests an association between CIPO and SIBO [5]. A study from Chile reported a 60% prevalence of hydrogen‑positive SIBO in patients with CIPO [6]; however, methane was not assessed. Recent advances in breath testing enable simultaneous measurement of hydrogen (H₂) and methane (CH₄), gases with contrasting effects on intestinal motility. Excessive methane production has been linked to delayed transit, leading to the adoption of the term “intestinal methanogen overgrowth” (IMO) [7]. Despite these developments, data on the prevalence and clinical characteristics of SIBO subtypes (H₂‑SIBO vs. IMO) in CIPO remain scarce, particularly in Asian populations [8]. Furthermore, most prior studies have not systematically compared gas phenotypes or explored their clinical correlates.

Given these gaps, we aimed to determine the prevalence and phenotype distribution of SIBO in Japanese patients with CIPO using glucose breath testing (GBT) in accordance with North American Consensus criteria. We also sought to describe associated clinical features and interpret our findings in the context of existing literature.

This article was previously posted to the Research Square preprint server on July 2, 2025 (Version 1; DOI: 10.21203/rs.3.rs-6928176/v1).

## Materials and methods

Study design and participants

We conducted a single‑center cross‑sectional study at Niigata University, Niigata, Japan (April 2019-March 2022). The Institutional Review Board approved the study protocol (approval no. 2019‑0226), and all participants provided written informed consent. The inclusion criteria were age ≥18 years and a diagnosis of CIPO according to national criteria [9], including radiologic evidence of bowel dilatation without mechanical obstruction. CIPO was classified as primary (idiopathic) or secondary (e.g., systemic sclerosis, drug‑induced, and pregnancy‑related). Ten outpatients with CIPO and 10 healthy controls (asymptomatic, no major comorbidities or gastrointestinal surgery) were enrolled. The study adhered to the Declaration of Helsinki and Japanese ethical guidelines for medical research.

Breath testing protocol

**Figure 1:**
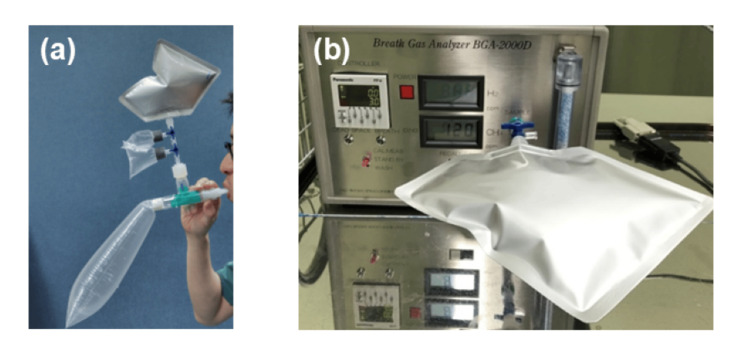
Glucose breath test procedure and equipment for SIBO diagnosis a Actual breath test measurement scene. b Equipment required for SIBO breath testing. SIBO: small intestinal bacterial overgrowth

In this study, a breath test was performed to diagnose SIBO. For accurate diagnosis, bread, pasta, and noodles, which increase hydrogen production, were not allowed for 24 hours before the breath test. In addition, oral intake of water or tea was not allowed for 12 hours before the test. Regular medications were administered two hours before the test, if necessary, and participants were advised to avoid drinking, exercising, and smoking during this period. Oral rinsing was performed to avoid the metabolism of sugar substrates by oral bacteria. Breath measurements (hydrogen and methane concentrations) were performed three times before sugar substrate loading, and the average score was used as the baseline. Subsequently, breath measurements were performed every 15 minutes after loading and up to 120 or 180 minutes. The sugar substrate used was 50 g of glucose. Exhaled hydrogen and methane concentrations were measured using a BGA2000D instrument (Laboratory for Expiration Biochemistry Nourishment Metabolism Co., Ltd., Nara, Japan) (Fig. 1a, 1b).

Diagnostic criteria and outcomes

According to the North American consensus [10,11], the following two diagnostic criteria for SIBO were used: a rise of ≥ 20 ppm from baseline in hydrogen by 90 minutes and a level of ≥10 ppm in methane. We have previously used these criteria to report the association between patients with cirrhosis and SIBO [12-14]. ΔH2 in Table 1 refers to the increase in hydrogen concentration from the baseline, and the CH4 peak value represents the highest methane concentration. Furthermore, cases with elevated hydrogen levels were defined as H-SIBOs, while those with elevated methane levels were defined as IMO, as previously reported [15]. In this study, both hydrogen- and methane-positive cases were included in the positive group for each sub-analysis. Exclusion criteria included recent antibiotic or probiotic use within four weeks, prior intestinal surgery (except colostomy), and severe comorbidities. No blinding was applied during data interpretation.

Statistical analysis

For data presented as proportions, 95% confidence intervals (CIs) were computed using the Clopper-Pearson exact method. The risk difference (RD) and its 95% CI were estimated using the Newcombe method (based on the Wilson score intervals). Risk ratios (RRs) were analyzed used the Katz log method; for zero cells, the Haldane-Anscombe continuity correction (0.5) was applied. Odds ratios (ORs) and 95% CIs used exact (Baptista-Pike) methods. Continuous variables are presented as the mean ± standard error, and categorical variables are expressed as counts and percentages. Given the small sample size (n = 10 per group) and zero cells, Fisher’s exact tests (two‑sided) were used for categorical comparisons, and Haldane-Anscombe continuity‑corrected odds ratios were calculated for zero‑cell tables. Mann-Whitney U tests were applied for exploratory comparisons of continuous variables, and U statistics with exact two-sided p-values are reported in the Results section. Statistical significance was set at p<0.05; however, results are interpreted cautiously due to limited power. Statistical analyses were performed using IBM SPSS Statistics for Windows, version 24.0 (IBM Corp., Armonk, N.Y., USA). An anonymized dataset and analysis templates are openly available to facilitate replication (DOI: 10.5281/zenodo.16910802).

## Results

The study included 10 patients with CIPO, comprising four males and six females, with a mean age of 48.1 ± 13.6 years and a mean duration of illness of 32.7 ± 35.5 months. The mean body mass index (BMI) of patients with CIPO was 16.9 ± 2.5 kg/m², and eight of the 10 individuals were underweight (BMI < 18.5), according to the Japan Society for the Study of Obesity (Table 1). Conversely, the mean age and BMI of the healthy controls were 31.6 ± 4.2 years and 22.6 ± 2.5 kg/m², respectively. The characteristics of CIPO were secondary and primary in seven and three cases, respectively. The underlying diseases associated with secondary CIPO included systemic sclerosis (one case), familial Mediterranean fever (one case), polymyositis (one case), Hashimoto’s disease (one case), pregnancy (two cases), and schizophrenia (Clozapine drug-induced) (one case). The affected sites were the small and large intestines in seven and three cases, respectively.

**Table 1:** Summary of patient characteristics. BMI: body mass index; SIBO: small intestinal bacterial overgrowth; CIPO: chronic intestinal pseudo-obstruction; ΔH2 refers to the increase in hydrogen concentration from the baseline; '+' indicates positive and '−' indicates negative.

Case (No.)	Age (years)	Sex	BMI (kg/m2)	SIBO	ΔH2 (ppm)	CH4 peak value (ppm)	Primary complaint	Duration of illness (months)	CIPO	Affected area	Other diseases	Past abdominal surgery
1	48	F	14.95	+	21.3	3	Bloating, diarrhea, nausea	10	Secondary	Small intestine	Systemic sclerosis	None
2	55	F	15.6	+	21	3	Abdominal pain, bloating	38	Secondary	Colon	Familial Mediterranean fever	None
3	72	M	13.3	+	105.7	3	Abdominal pain, nausea	113	Secondary	Small intestine	Polymyositis	None
4	62	M	16.3	+	96.4	1	Abdominal pain, bloating	18	Secondary	Small intestine	Hashimoto disease	Appendectomy
5	31	F	19.1	+	78	9	Abdominal pain, bloating, diarrhea	19	Secondary	Small intestine	Pregnancy	None
6	29	M	17.8	-	19	0	Bloating, constipation	6	Primary	Small intestine	Autism spectrum disorder	None
7	36	F	14.6	-	-17	1	Bloating	21	Primary	Colon	Pustular psoriasis	Colostomy (for CIPO)
8	40	F	17.3	-	-14	2	Bloating, constipation	88	Secondary	Small intestine	Pregnancy	None
9	61	F	17.7	-	2	0	Bloating, constipation	6	Secondary	Small intestine	Schizophrenia	Colostomy (for cancer)
10	47	M	22.4	-	8	1	Bloating	8	Primary	Colon	Autism spectrum disorder	Colectomy (for CIPO)

Representative case

**Figure 2:**
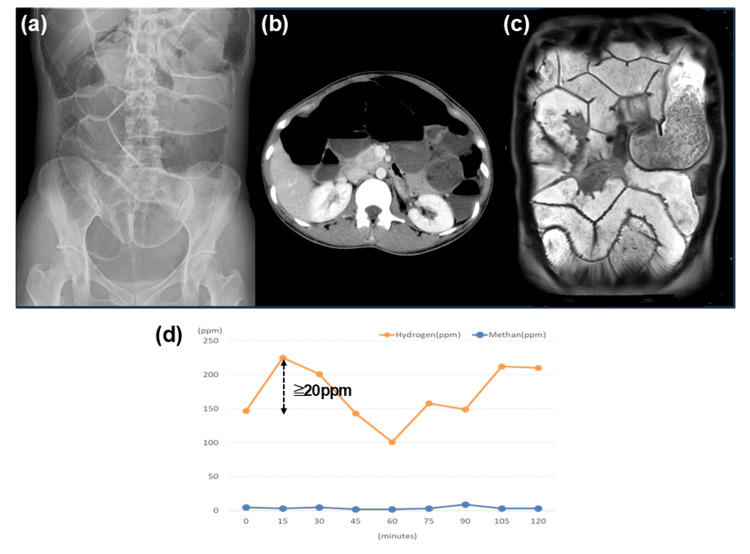
Radiologic and functional imaging findings in CIPO with hydrogen-positive SIBO (case 5) a Abdominal radiograph: marked distension of the intestine by intestinal gas is observed. Small intestinal gas occupies the largest part. b Abdominal CT: markedly dilated intestinal loops and air-fluid levels are seen. c Cine-MRI: small bowel dilatation and reduced peristalsis are seen. d: A breath test confirmed the diagnosis of hydrogen-type SIBO according to the North American Consensus. SIBO: small intestinal bacterial overgrowth; CIPO: chronic intestinal pseudo-obstruction; CT: computed tomography; MRI: magnetic resonance imaging

Abdominal radiographs, computed tomography, cine-magnetic resonance imaging (MRI), and breath test results for a representative case of CIPO are shown in Figure 2. This is a case of secondary intestinal pseudo-obstruction that developed in a 31-year-old woman after pregnancy and childbirth (case 5). The symptoms included abdominal pain, bloating, and diarrhea. Abdominal radiography revealed significant gaseous distension, predominantly in the small intestine. Abdominal radiographs and computed tomography demonstrated markedly dilated intestinal loops with air-fluid levels (Fig. 2a, 2b). Cine-MRI revealed small bowel dilatation and reduced peristalsis (Fig. 2c). A breath test confirmed the diagnosis of hydrogen-type SIBO, according to the North American Consensus (Fig. 2d).

In our study, hydrogen/methane breath testing showed that five of the 10 cases were SIBO-positive using the North American Consensus (six of the 10 using the Rome Consensus9), and all cases were associated with SIBO-phenotype hydrogen type. By contrast, when SIBO was measured in 10 healthy controls (male-to-female ratio 8:2, average age 31.6 ± 4.22 years, average BMI 22.7 ± 2.46, no symptoms, and no history of underlying diseases or surgery), only one case was diagnosed as having IMO, and no participant was diagnosed with hydrogen SIBO.

Primary outcome

**Figure 3:**
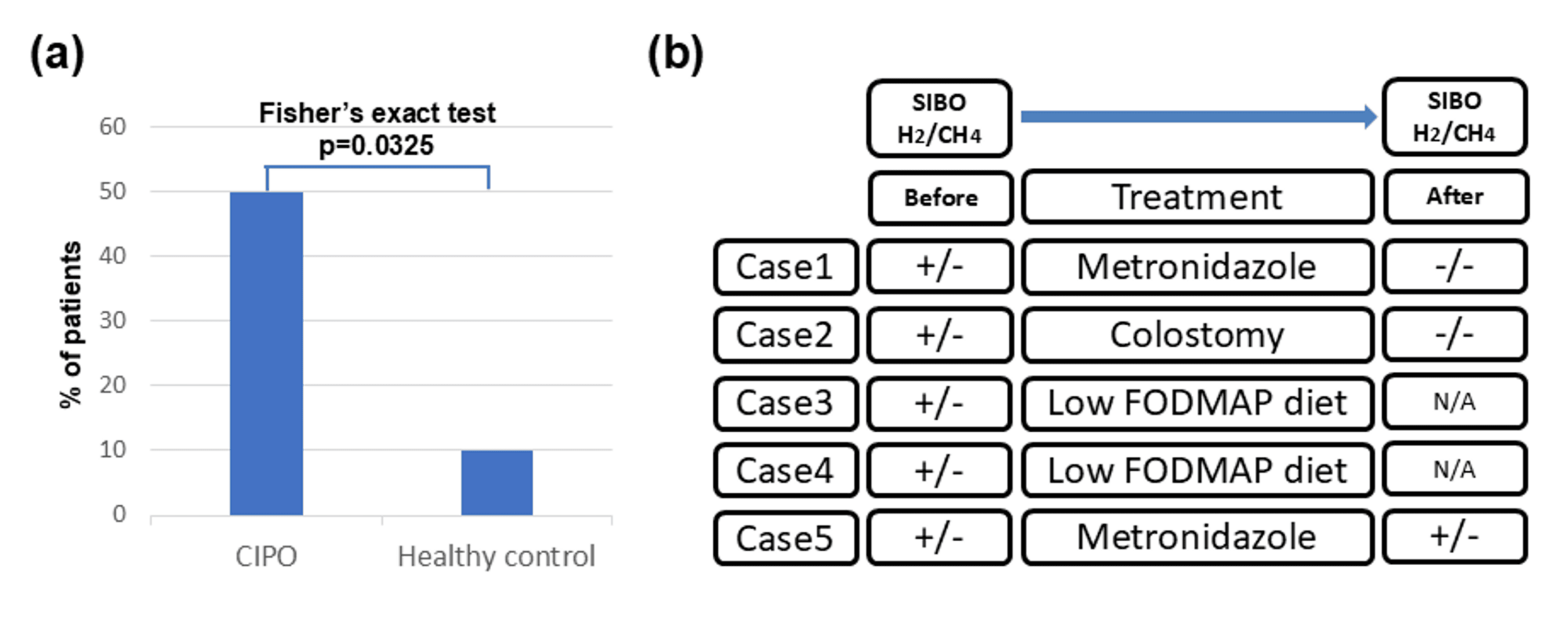
Prevalence of SIBO in CIPO and treatment flow for SIBO-positive patients a Prevalence of overall SIBO (H₂ or CH₄) in patients with CIPO and healthy controls (Fisher’s exact test for H₂-SIBO; CIPO n = 10, controls n = 10). b Flowchart before and after treatment for SIBO-positive patients. SIBO: small intestinal bacterial overgrowth; CIPO: chronic intestinal pseudo-obstruction; FODMAP: fermentable oligosaccharides, disaccharides, monosaccharides, and polyols

Hydrogen-positive SIBO (H₂-SIBO) was observed in 5/10 patients with CIPO and 0/10 controls (Fisher’s exact test, two-sided p = 0.0325; continuity-corrected OR = 21, 95% CI: 1.8-243.8) (Fig. 3a). The risk difference was 50.0% (95% CI 11.7-76.3%), with an exact OR of 20.0 (95% CI 0.90-443.50) and an RR (Haldane-Anscombe adjusted) of 11.0 (95% CI 0.69-175.87). For overall SIBO (H₂ or CH₄), the prevalence was 5/10 vs. 1/10 (p=0.1409), with a risk difference of 40.0% (95% CI −0.24-67.59%), exact OR 9.0 (95% CI 0.81-100.14), and RR 5.0 (95% CI 0.70-35.50).

Secondary outcomes

Overall SIBO (H₂ or CH₄ positive) occurred in 5/10 CIPO vs. 1/10 controls (p = 0.1409). No patient with CIPO met IMO criteria; one control was methane-positive. Baseline H₂/CH₄ values and full time-course data are provided in Zenodo (DOI: 10.5281/zenodo.16910802).

Among patients with CIPO, those with H₂-SIBO tended to have lower BMI (15.6 kg/m² vs. 18.0 kg/m²) and more frequent diarrhea (40% vs. 0%) than SIBO-negative patients, although these differences were not statistically significant; other symptoms (abdominal pain, bloating, nausea) were common in both groups.

Exploratory comparisons using exact Mann-Whitney U tests showed that ΔH₂ by 90 min tended to be higher in CIPO than in controls (median 20.0 ppm (IQR 3.5-63.8) vs 4.0 (2.3-4.8); U = 70.5, p = 0.130), whereas CH₄ peak did not differ significantly (1.5 (1.0-3.0) vs 1.0 (0.0-1.0); U = 68.5, p = 0.153). Within the CIPO cohort, H₂‑SIBO-positive patients had markedly higher ΔH₂ by 90 min (78.0 (21.3-96.4) vs 2.0 (-14.0-8.0); U = 25.0, p = 0.008) and higher CH₄ peak (3.0 (3.0-3.0) vs 1.0 (0.0-1.0); U = 23.0, p = 0.032), whereas age and BMI differences were not significant.

Treatment observations

These observations are anecdotal and interpreted cautiously. Of the five SIBO-positive patients, case 2 underwent colostomy, and cases 1 and 5 were treated with metronidazole, which improved their symptoms (Fig. 3b). After treatment, SIBO was measured, and two (cases 1 and 2) of the three patients tested negative. Cases 3 and 4 were treated with a low fermentable oligosaccharides, disaccharides, monosaccharides, and polyols (FODMAP) diet; however, SIBO testing was not performed after treatment due to patient preference.

## Discussion

To date, limited reports have examined the relationship between CIPO and SIBO (Table 2).

**Table 2:** Summary of previous reports on CIPO and SIBO. SIBO: small intestinal bacterial overgrowth; CIPO: chronic intestinal pseudo-obstruction; LBT: lactulose breath test; GBT: glucose breath test; FMT: fecal microbiota transplantation; RFX: rifaximin; MNZ: metronidazole

Study (No.)	Authors	Study year	Country	Mode of diagnosis for SIBO	Measured gas type	Diagnostic criteria for SIBO	Patients with CIPO N	H2-SIBO in CIPO patients N (%)	CH4-SIBO in CIPO patients N (%)	Treatment /notes
1	Pérez et al. [6]	2014	Chile	LBT	H2	Original (described in the text)	40	24 (60)	N/A	None
2	Lili et al. [16]	2017	China	LBT	H2	Rome Consensus	9	7 (78)	N/A	FMT
3	Khan et al. [8]	2021	USA	GBT	H2 and CH4	North American Consensus	38	9 (24)	20 (53)	CH4
4	Okubo et al. [9]	2024	Japan	GBT	H2	Rome Consensus	12	8 (67)	N/A	RFX
5	Ours	2025	Japan	GBT	H2 and CH4	North American Consensus	10	5 (50)	0 (0)	CH4, MNZ

In 2014, Pérez et al. reported a 60% prevalence of SIBO in 40 patients with CIPO using the lactulose hydrogen breath test (LBT), although methane was not assessed and diagnostic criteria differed from current standards [6]. Lili et al. later reported a 78% prevalence using Rome Consensus thresholds [16,17]. However, the LBT has been criticized for overdiagnosis due to rapid colonic fermentation, and the North American Consensus currently recommends GBT with defined cutoffs (ΔH₂ ≥ 20 ppm by 90 min; CH₄ ≥ 10 ppm) [10,18].

Recent studies using GBT have yielded variable results. Khan et al. (USA) found IMO in 53% of patients with CIPO, whereas H₂‑SIBO was not significantly different from controls [8]. In contrast, Okubo et al. (Japan) reported a 67% prevalence of H₂‑SIBO using the Rome criteria but did not measure methane [9]. Our re‑analysis adds to this literature using North American Consensus criteria and exact statistics in a Japanese cohort. We observed that H₂‑SIBO was more frequent in CIPO (50%) than in controls (0%), while IMO was uncommon, contrasting with the US study where IMO predominated. These discrepancies may reflect methodological differences (substrate, cutoffs), constipation phenotypes, and cultural or dietary factors, as methane production has been linked to slow transit and constipation [19-21]. The single methane‑positive control in our study had recently returned from the UK, suggesting environmental influences.

Clinically, H₂‑SIBO‑positive patients tended to have lower BMI and more frequent diarrhea, consistent with the physiological effects of hydrogen‑producing flora. However, these associations are descriptive and hypothesis‑generating. Treatment observations (metronidazole response in two cases and dietary modification in others) are anecdotal and not generalizable; randomized trials are needed to define optimal therapy [22-24].

Limitations include the small sample size, single‑center design, and lack of blinding, which may limit generalizability. The glucose breath test may underestimate distal small bowel overgrowth. Age/BMI mismatch between groups and reliance on breath testing as an indirect measure also constrain interpretation. Nevertheless, this study provides the first combined assessment of hydrogen and methane in Asian patients with CIPO using consensus‑based GBT.

## Conclusions

The findings of the present study suggest an association between CIPO and H₂‑SIBO in a Japanese cohort, while IMO was rare. However, these findings should be interpreted with caution given the small sample size, single-center design, and potential under-detection of distal overgrowth with glucose substrate. Further multicenter studies with larger cohorts and standardized protocols are warranted to confirm these observations. An anonymized dataset and analysis templates are openly provided to facilitate replication.
